# Asiatic acid attenuates lipopolysaccharide-induced injury by suppressing activation of the Notch signaling pathway

**DOI:** 10.18632/oncotarget.24542

**Published:** 2018-01-23

**Authors:** Xiong Yuyun, Cheng Xi, Yin Qing, Xia Lin, Rui Ke, Sun Bingwei

**Affiliations:** ^1^ Department of Clinical Laboratory, Affiliated Hospital of Jiangsu University, Zhenjiang, Jiangsu 212001, P.R. China; ^2^ Atom Bioscience and Pharmaceutical Co., Ltd., Zhenjiang, Jiangsu 212001, P.R. China; ^3^ Department of Burn and Plastic Surgery, Affiliated Hospital of Jiangsu University, Zhenjiang, Jiangsu 212001, P.R. China

**Keywords:** asiatic acid, lipopolysaccharide, Notch, sepsis

## Abstract

Sepsis is a severe multisystem disease with high mortality rates and limited treatment options. However, advances during the last decade have opened opportunities to develop novel therapeutic strategies. The Notch signaling pathway plays a critical role in inflammation, and its inhibition offers an avenue to treat inflammatory diseases, such as sepsis. Asiatic acid (AA), a triterpenoid isolated from Centella asiatica, reportedly exerts anti-oxidant, anti-tumor, and anti-inflammatory effects, but its mechanisms remain unclear. In our study, we found that AA decreased levels of interleukin-1β (IL-1β), IL-6, alanine aminotransferase and blood urea nitrogen in serum; attenuated liver, lung and kidney damage; and improved the survival among mice with experimental sepsis. AA also reduced lipopolysaccharide-stimulated expression of proinflammatory mediators, including nitric oxide, IL-1β and IL-6 in RAW 264.7 macrophages. Notably, we demonstrated for the first time that AA is a novel small molecule inhibitor of the Notch signaling pathway. Its effects include upregulation of Notch receptor (Notch3) and delta-like ligand (DLL4), inhibition of Notch3 binding to the IL-6 promoter and regulation of mitochondrial function. These novel effects of AA may provide new approaches and strategies for the treatment of inflammatory disorders.

## INTRODUCTION

The pathogenesis of sepsis is complex and multifactorial, however, lipopolysaccharide (LPS) from Gram-negative bacteria is one of the key causative factors. LPS triggers excess or inappropriate secretion of a variety of proinflammatory cytokines, such as tumor necrosis factor-α(TNF-α), interleukin 1β (IL-1β), IL-6, nitric oxide synthase (iNOS) and other effector enzymes [1]. These proinflammatory mediators induce tissue damage, which eventually results in the dysfunction of multiple organs and septic shock [2].

LPS can activate multiple signaling pathways, including nuclear factor κB (NF-κB), mitogen-activated protein kinase (MAPK) and Notch pathways [3]. More recently, there has been a greater appreciation for the importance of Notch in a variety of inflammatory conditions, including rheumatoid arthritis (RA), [4, 5] systemic lupus erythematosus [6] and bacterial and viral infections [7, 8]. In mammals, the Notch family consists of four Notch receptors (Notch1-4) and five Notch ligands, Delta-like ligands (Dll) 1, 3, and 4 and Jagged 1 and 2. It has been reported that the Notch signaling pathway is activated in both early septic shock patients and THP-1 cells in response to LPS challenges [9]. In activated macrophages, Notch leads to elevated expression of proinflammatory cytokines. Whereas, inhibition of Notch by a γ-secretase inhibitor, Notch silencing [1, 10] or genetic ablation of Csl [11] diminishes LPS-induced expression of proinflammatory genes. Moreover, Notch signaling directly regulates IL-6 mRNA expression in macrophages by associating with its promoter region [12]. Due to its association with inappropriately amplified inflammatory responses, the Notch signaling pathway offers a potential therapeutic approach to sepsis and other serious inflammatory diseases.

**Figure 1:**
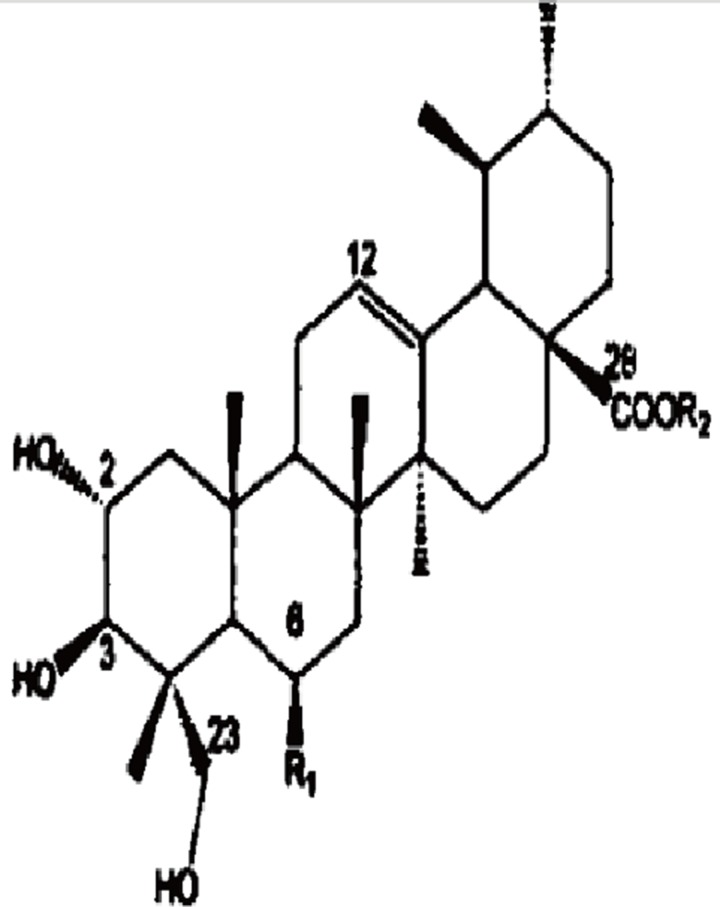
The chemical structure of AA

The treatment of sepsis relies heavily on various antibiotics, which, when overused or misused can result in antibiotic resistance bacteria. Asiatic acid (AA), a triterpenoid isolated from Centella asiatica (Figure 1), has been reported to exhibit anti-inflammatory effects in fulminant hepatitis [13], experimental colitis [14] and acute lung injury models [15]. AA has been reported to suppress the protein and mRNA levels of TNF-α, IL-1β and IL-6 by suppressing mitochondria-mediated NLRP3 inflammasome activation [14] or TLR4 expression and NF-kB activation [15]. In this study, we investigated the anti-sepsis effect of AA and its potential mechanisms in relation to Notch signaling.

## RESULTS

### Protective effect of AA against LPS-induced sepsis in mice

**Figure 2:**
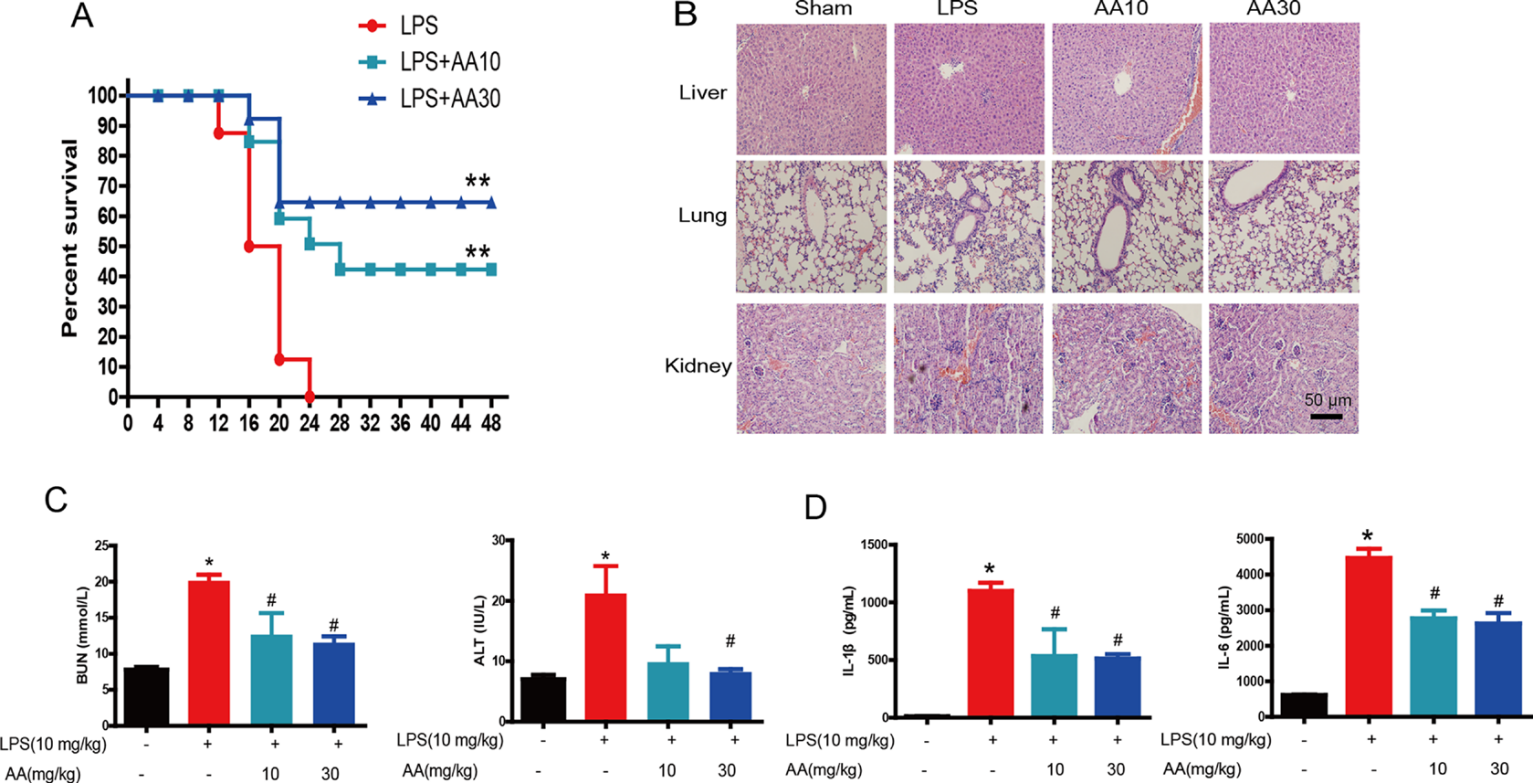
Protective effect of AA against LPS-induced sepsis in mice BALB/c mice were administered LPS (10 mg/kg) and AA (10 and 20 mg/kg). (**A**) Survival rate. (**B**) Photomicrographs of representative mouse lung, liver and kidney sections with H&E staining (original magnification ×200). (**C**) Serum levels of ALT and BUN were measured 4 h after LPS injection. (**D**) Serum cytokine levels of IL-1β and IL-6 were measured by ELISA 4 h after LPS injection. Data are shown as mean ± SEM (*n* = eight mice in each group), and each experiments were duplicated for 3 times. ^*^p < 0.05 versus sham control, ^#^*p* < 0.05 versus LPS control.

To measure the anti-inflammatory effects of AA against LPS-induced sepsis *in vivo*, BALB/c mice were administered LPS (10 mg/kg) and AA (10 and 30 mg/kg). No mice survived at 24 hrs after LPS injection (Figure 2A). Notably, 30 mg/kg AA significantly improved mice survival for up to 60%. Moreover, the sections stained with hematoxylin & eosin (H&E) showed neutrophil infiltration, cellular necrosis, alveolar wall thickening and endothelial cell swelling in organs after LPS treatment. In contrast, AA markedly reduced the extent of tissue damage and neutrophil infiltration (Figure 2B). The protective effect of AA was further confirmed by the results that AA-treated mice had a reduction of serum alanine aminotransferase (ALT) and blood urea nitrogen (BUN) levels compared to LPS-infused mice (Figure 2C). Furthermore, ELISA also demonstrated that AA inhibited LPS-induced release of IL-1β and IL-6 (Figure 2D).

### Effect of AA on cell viability in RAW264.7 cells

**Figure 3:**
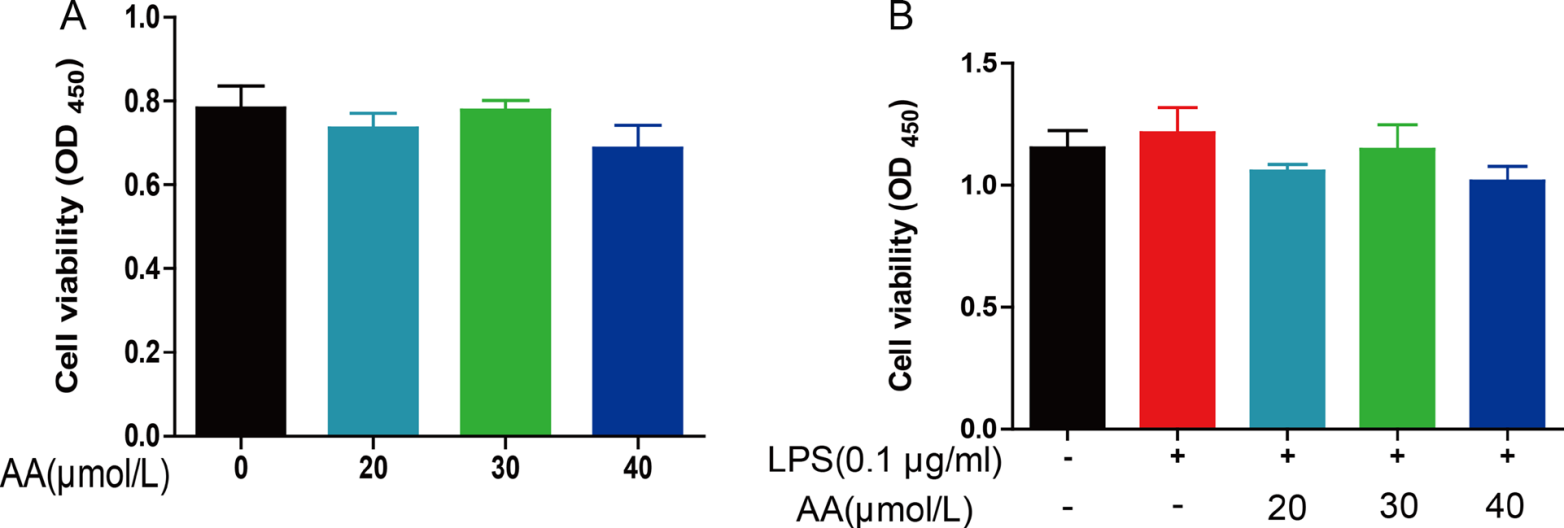
The effect of AA on cell viability of RAW264.7 RAW264.7 cells were treated with various concentrations of AA in the absence (**A**) or presence of LPS (**B**) for 48 h and then CCK-8 was used to detach cell viability. Data are shown as means ± S.D (*n* = 5), and each experiments were duplicated for 3 times.

To determine the effects of AA on cell viability, RAW264.7 cells were exposed to AA for 48 hrs in the absence or presence of LPS. Cell survival was assessed by CCK-8 assay. Without LPS stimulation, AA (20–40 μM) failed to impact viability of RAW264.7 cells (Figure 3A). Similar effects were observed under LPS challenge (Figure 3B). 20–40 μM of AA was used in the subsequent *in vitro* experiments.

### AA inhibited LPS-induced production and transcription of proinflammatory mediators in RAW264.7 cells

**Figure 4:**
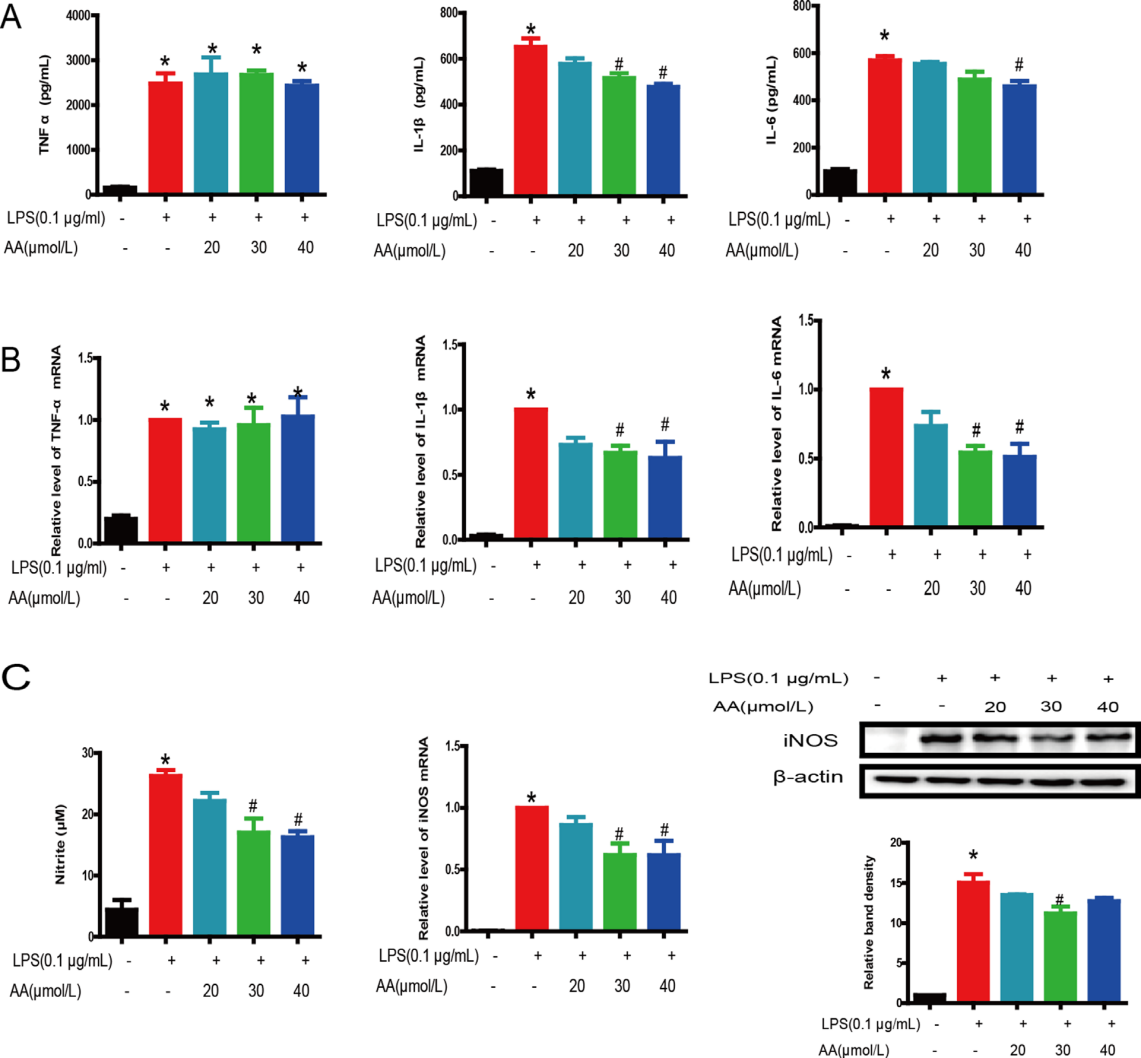
Inhibition of LPS-induced proinflammatory mediator production by AA in RAW264.7 cells (**A**) Levels of TNF-α, IL-1β and IL-6 in the cell culture medium were determined at 48 h after LPS and AA stimulation. (**B**) Gene expression levels of TNF-α, IL-1β and IL-6 mRNA were determined by real-time quantitative PCR after AA and LPS stimulation. β-Actin was used as a control. (**C**) The levels of NO in the cell culture medium and expression of iNOS in cells were determined 48 h after AA and LPS stimulation. Data are shown as mean ± SD (*n* = 5), and each experiments were duplicated for 3 times. ^*^p < 0.05 versus control, ^#^*p* < 0.05 versus LPS group.

Excessive and uncontrolled production of proinflammatory mediators, including cytokines and effector enzymes, have been implicated in serious inflammatory diseases, such as atherosclerosis, RA and sepsis [3, 16, 17]. The effect of 20–40 μM AA on the production of TNF-α, IL-1β and IL-6 in the culture medium of RAW264.7 cells was detected by ELISA. AA attenuated LPS-induced IL-1β and IL-6 augmentation in RAW264.7 cells in a dose-dependent manner (Figure 4A). Real-time PCR indicated that AA markedly inhibited LPS-induced expression of IL-1β and IL-6 in a dose-dependent manner (Figure 4B). However,

AA (≤ 40 μM) did not impact TNF-α release or mRNA induction. To examine the suppressive effect of AA on NO production in LPS-stimulated RAW264.7 cells, we used the Griess method to measure nitrite. We found that LPS induced a large amount of NO production, and AA suppressed the formation of NO in a dose-dependent manner. In addition, mRNA and translation levels of iNOS showed similar results (Figure 4C).

### Effect of AA on LPS-induced activation of NF-κB and MAPK signaling in RAW264.7 cells

**Figure 5:**
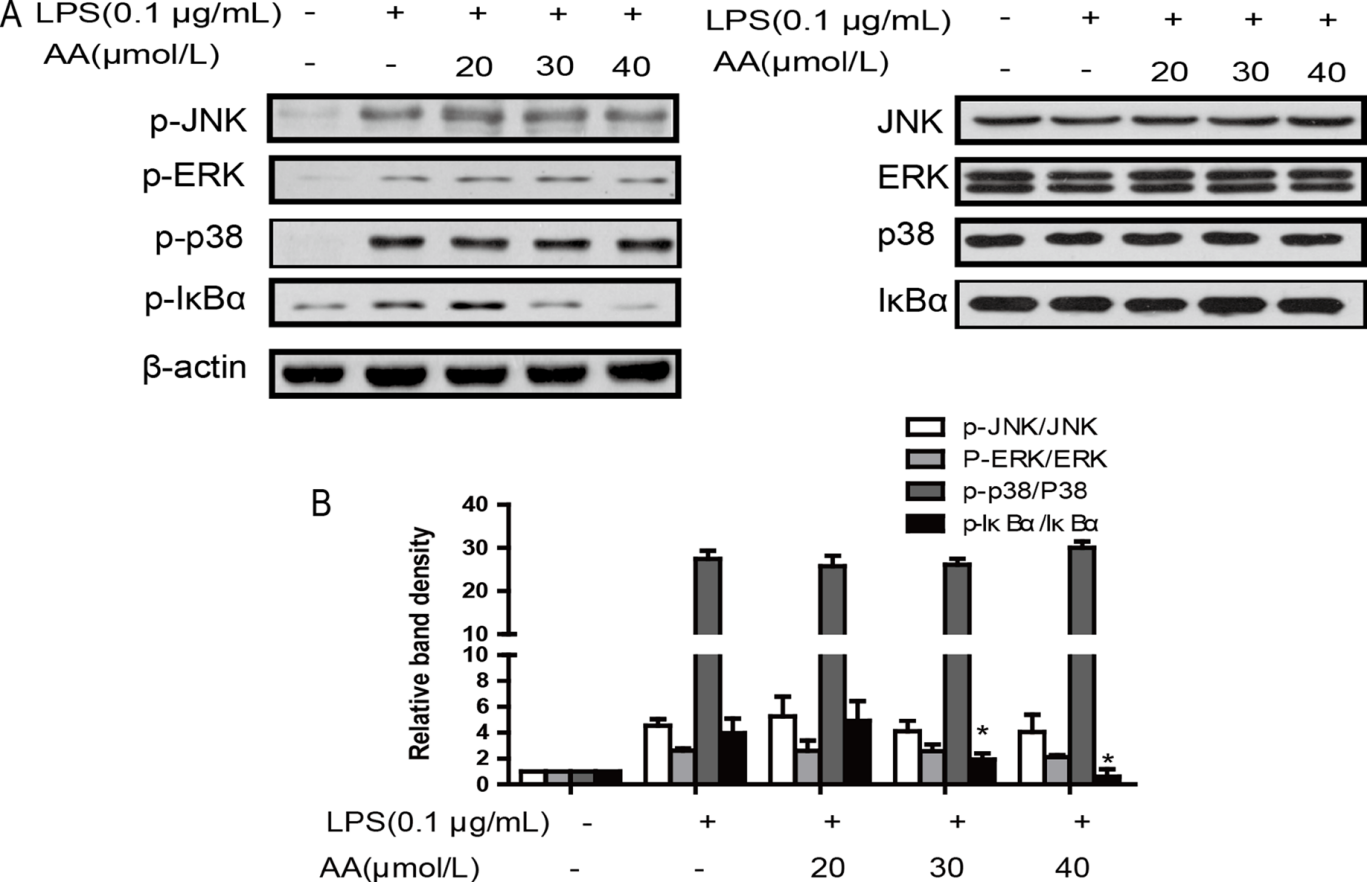
Effects of AA on LPS-induced activation of MAPKs and NF-κB in RAW264.7 cells (**A**) Cells were treated with AA in the absence or presence of LPS for 24 h. The protein levels of total and phosphorylated p38, JNK, ERK and IκBα were determined at least 3 times. (**B**) Summary data of (a) are expressed as a histogram of mean ± SD of 3 independent experiments. ^*^p < 0.05 versus control, ^#^*p* < 0.05 versus LPS group.

Evidence indicates that NF-κB and MAPK can regulate the transcription of proinflammatory cytokine genes [24]. We hypothesized that AA may inhibit NF-κB and MAPK signaling pathways upon response to LPS. As shown in Figure 5, AA inhibited the LPS-induced phosphorylation of IκBα without any effect to JNK, ERK or P38. These findings suggest that AA might suppress LPS-induced NF-κB activation but not LPS-induced MAPK signaling.

### AA directly reduced LPS challenged activation of Notch in RAW264.7 cells

**Figure 6:**
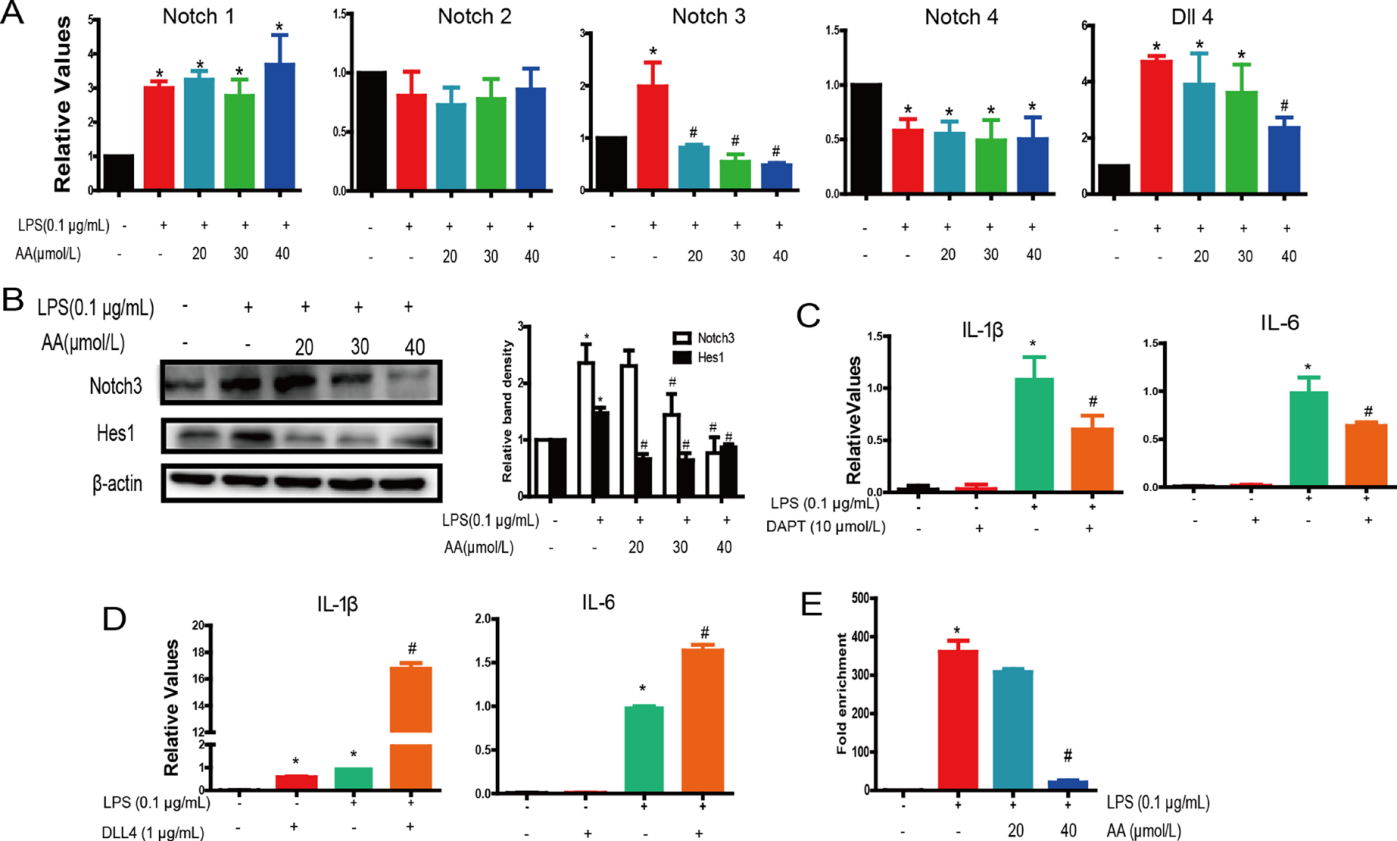
AA directly reduced LPS-induced activation of Notch in RAW264.7 cells (**A**) Cells were treated with various concentrations of AA in the absence or presence of LPS for 48 h, followed by analysis of Notch receptors and Dll4 expression by PCR. (**B**) Protein expression of Notch3 and Hes1 in AA and LPS treatment of Raw264.7 cells. (**C**) RAW264.7 cells were cultured in the presence of LPS (0.1 μg/mL) in addition to for 48 h. IL-1β and IL-6 expression was determined by qPCR. (**D**) RAW264.7 cells were cultured in the presence of LPS (0.1 μg/mL) in addition to Dll4 fusion protein (1 μg/mL) for 48 h. IL-1β and IL-6 expression were determined by qPCR. (**E**) Association of Notch3 and IL-6 gene promoter region was measured in the immunoprecipitate by quantitative PCR relative to that for input DNA. ^*^p < 0.05 versus control, ^#^*p* < 0.05 versus LPS control. Data are shown as mean ± SD (*n* = 5), and each experiments were duplicated for 3 times.

Activation of Notch signaling triggered upregulation of proinflammatory responses in LPS-challenged macrophages. We next determined by real-time PCR the effect of AA on the mRNA expression of Notch receptors and ligands in LPS-challenged RAW264.7 cells. AA markedly inhibited LPS-induced upregulation of Notch3 and Dll4. Nevertheless, there were no effects on the expression of Notch 1, 2 or 4 (Figure 6A). The translation levels of Notch 3 and hes1, a target gene of Notch, showed similar results (Figure 6B).

To validate the role of AA in suppressing Notch signaling in RAW264.7 cells, we used DAPT, a Notch signaling inhibitor, in LPS-challenged RAW264.7 cells. DAPT significantly inhibited IL-1β and IL-6 expression in response to LPS (Figure 6C). Moreover, we also examined the effect of a Dll4 murine recombinant fusion protein on the augmentation of cytokine expression in LPS-treated macrophages. When pre-treated with Dll4 protein, IL-1β and IL-6 production increased versus the LPS group (Figure 6D). A previous study has shown that Notch is associated with the IL-6 promoter in LPS/interferon-γ-activated RAW264.7 cells, but not in unstimulated cells [12]. We, therefore, carried out a Chromatin immunoprecipitation (ChIP) assay to test whether AA inhibited Notch3 binding to the IL-6 promoter. High association of Notch3 with the IL-6 promoter was detected after LPS stimulation, which was suppressed by AA (Figure 6E). These results suggest that Notch is an important target of AA in regulating proinflammatory mediator expression in LPS-treated RAW264.7 cells.

### AA inhibited ROS generation and mitochondria dysfunction

**Figure 7:**
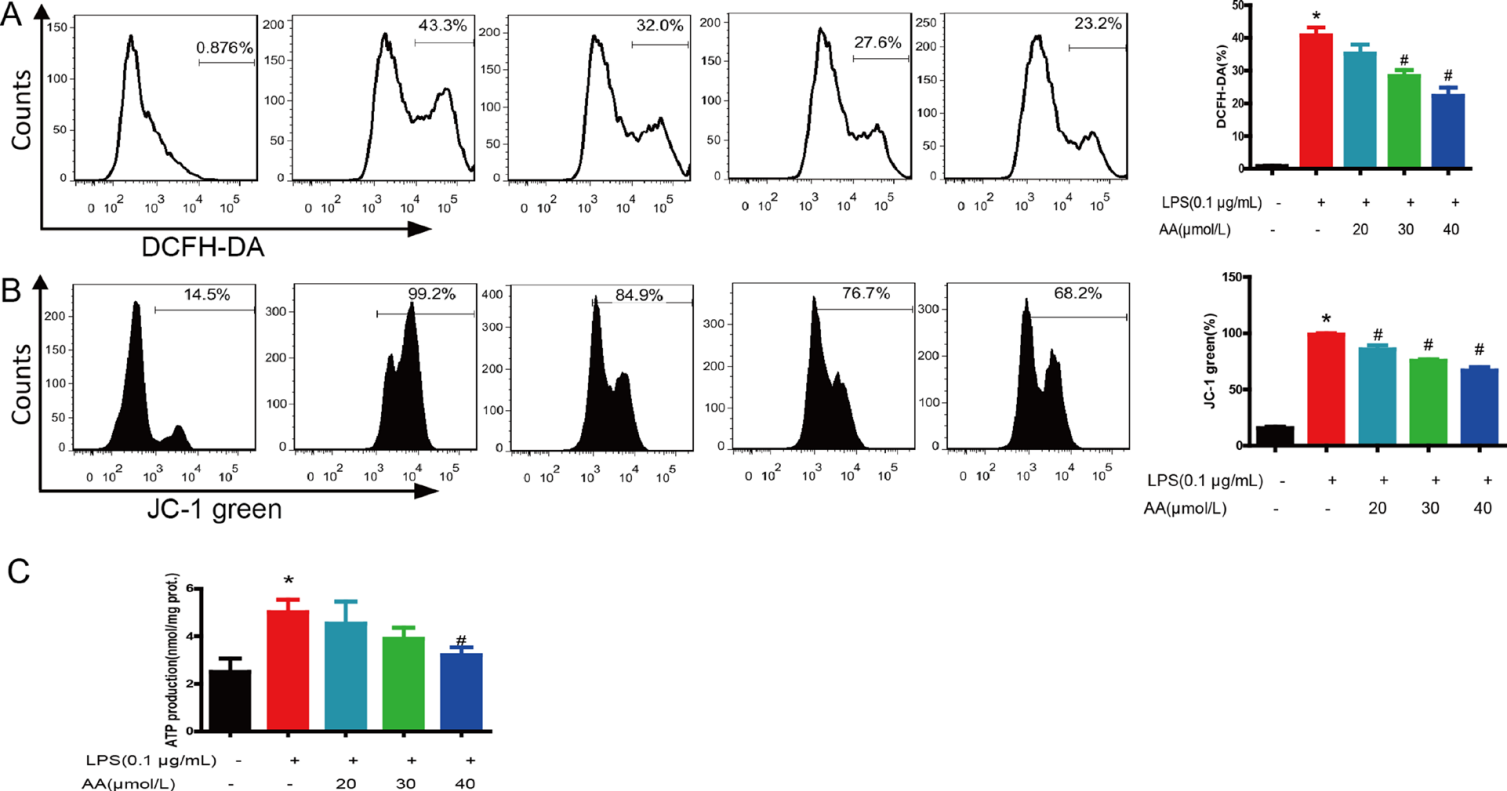
AA treatment reduced ROS generation and recovered mitochondrial function Cells were treated with various concentrations of AA in the absence or presence of LPS for 48 h, and then stained with DCFH-DA (**A**) or JC-1 (**B**) and analyzed by flow cytometry. (**C**) ATP synthesis was measured by a luciferin–luciferase assay as described in “Materials and Methods”. ^*^p < 0.05 versus control, ^#^*p* < 0.05 versus LPS control. Data are shown as mean ± SD (*n* = 5), and each experiments were duplicated for 3 times.

Our previous study demonstrated that AA could directly interact with mitochondria and prevent LPS-induced mitochondrial dysfunction [18]. LPS treatment caused ROS elevation (Figure 7A), mitochondrial membrane potential (MMP) collapse (Figure 7B) and ATP reduction (Figure 7C). AA treatment significantly scavenged ROS and restored MMP and ATP production (Figure 4).

## DISCUSSION

**Figure 8:**
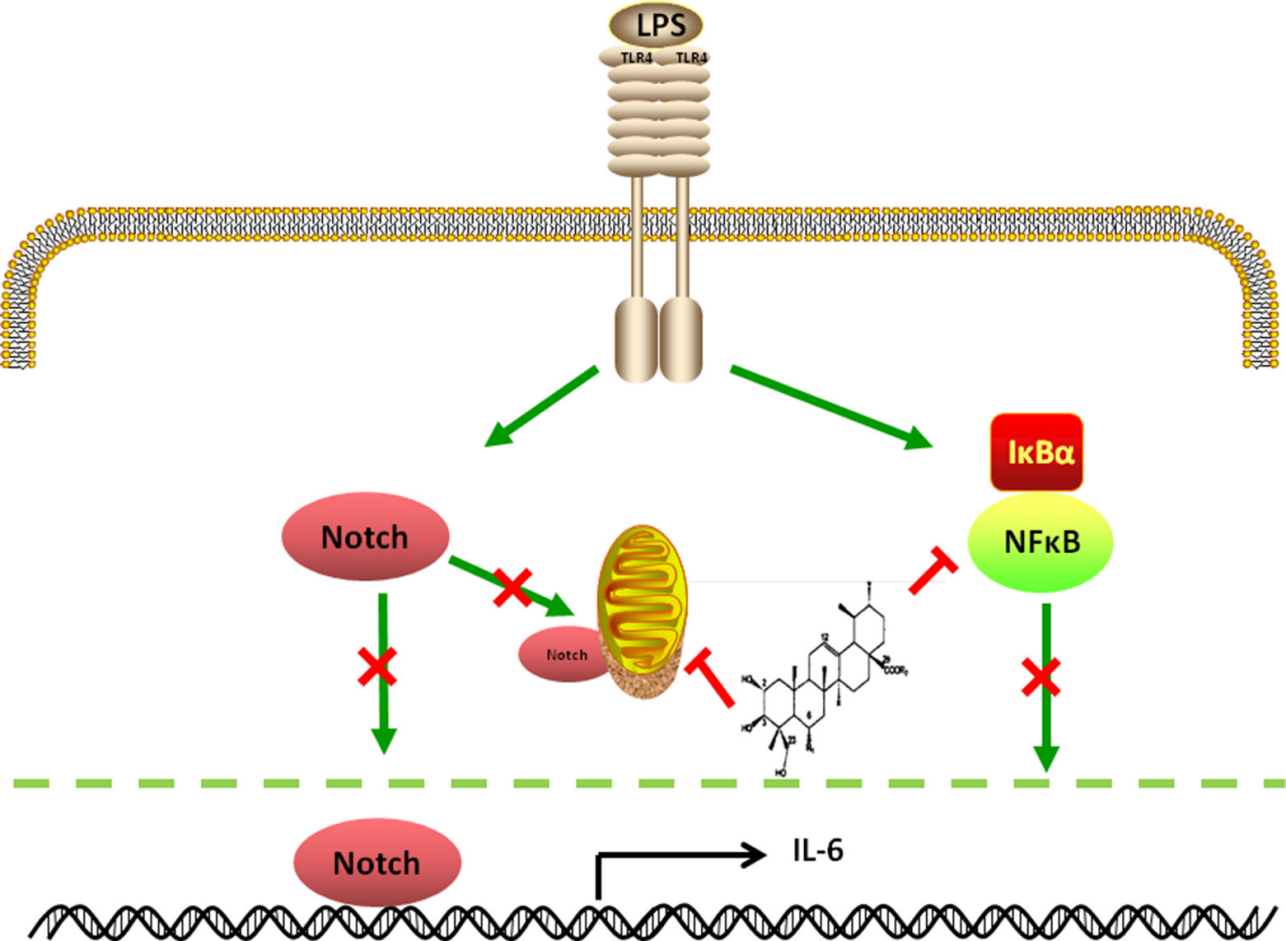
Illustration for the mechanism underlying inhibition of Notch signaling pathway by AA against LPS-challenged macrophages and sepsis in mice AA could inhibit ROS generation and mitochondrial dysfunction, followed by suppressing the NF-κB and Notch signaling pathways, and finally refrain cytokine production.

Here, for the first time, we demonstrate that AA is a novel small molecule inhibitor of the Notch signaling pathway and exerted anti-sepsis effects by preventing Notch3 from binding to the IL-6 promoter and regulating mitochondrial function (Figure 8).

LPS, a well-characterized pathogen-associated molecular pattern, has been found in the outer leaflet of the outer membrane of most of the Gram-negative bacteria. It has been recognized as a well-accepted and validated model of sepsis characterized by excess or inappropriate production of pro-inflammatory cytokines, elevated mortality and tissue damage [19]. In our present study, we found that LPS increased the mortality of sepsis in mice, which could be blocked by pre-treatment with AA. Meanwhile, AA improved organ function and reduced organ damage. Additionally, AA attenuated LPS-induced production of cytokines, such as iNOS, IL-1β and IL-6 in RAW264.7 macrophage cells and mice with experimental sepsis. However, TNF-α production and mRNA induction was unaffected by AA *in vitro*. It is possible that multiple signal pathways activated by LPS are involved in the regulation of TNF-α expression. Consistent with our data, previous studies have also reported that AA has anti-inflammatory properties against dextran sulfate sodium-induced murine experimental colitis and LPS-induced acute lung injury. However, the underlying mechanism remains unclear [13, 15, 20].

The NF-κB signaling pathway is one of the most important signaling pathways involved in LPS stimuli. Activated NF-κB signaling has been found in various responses, leading to host defense through rapid induction of proinflammatory cytokines, chemokines and adhesion molecules [21]. The activation of NF-κB is regulated by cellular signaling, including MAPK [22] and Notch [1]. MAPKs are a highly conserved family of protein serine/threonine kinases and include the p38, ERK1/2 and JNK subgroups [23]. In the present study, we found that LPS induced activation of NF-κB and MAPK pathways. Nevertheless, AA inhibited the LPS-induced phosphorylation of IκBα without any effect on JNK, ERK or P38. In contrast, Yun et al. found that the anti-inflammatory properties of AA might result from downregulation of NF-κB activation via suppression of IKK and MAPK (p38, ERK1/2 and JNK) phosphorylation in RAW 264.7 cells. The paradoxical results may be due to the different concentration of AA used in study.

Recently, the crucial role of Notch signaling implicated in the inflammatory responses has attracted more researchers interest. Palaga, T. et al. found that Notch signaling activated upon stimulation of macrophages through the Toll-like receptor (TLR) signaling cascade, which in turn regulated gene expression patterns involved in pro-inflammatory responses [10]. Meanwhile Dll4 increased in macrophages exposed to proinflammatory stimuli such as LPS, IL-1β, or minimally-modified low-density lipoprotein in a TLR 4- and NF-κB-dependent fashion [24]. Further study demonstrated that Notch-RBP-J and TLR signaling are integrated at the level of IRF8 protein synthesis and identify a mechanism by which heterologous signaling pathways can regulate TLR-induced inflammatory macrophage polarization [25]. Similarly, our results also showed that the expression of various Notch pathway components including Notch 1, 3 and Dll4 were elevated in LPS stimulated macrophages.

Notch’s role in the regulation of inflammatory cytokines and enzymes responsible for inflammation makes it an attractive drug target. Several small molecular inhibitors targeting Notch have been developed and evaluated in animal models of inflammatory diseases [10, 12, 26]. Similarly, our results demonstrated AA-mediated attenuation of proinflammatory mediators was associated with down-regulation of the transcription of Notch3 and Dll4 *in vitro*. Meanwhile, treatment with a Notch selective inhibitor, DAPT, inhibited expression of proinflammatory mediators. Conversely, pretreatment with Dll 4 augmented IL-1β and IL-6 production. Notch signaling could directly regulate IL-6 mRNA expression by associating with the promoter region of IL-6 [12]. Our results also showed that AA suppressed the Notch3 binding to IL-6 promoter. These results demonstrated that Notch signaling may be directly involved in the anti-inflammatory effect of AA.

Recent study showed that ROS generated by mitochondrial respiration are important for LPS-driven production of several proinflammatory cytokines [27]. Meanwhile, Notch1 could translocate to mitochondria and couple upregulation of M1 genes with upregulation of mitochondrial oxidative phosphorylation and ROS [28]. Here, we showed that AA treatment effectively prevented LPS-induced collapse of the MMP, ROS generation and ATP reduction. These results coincided with our previous study that AA could directly interact with mitochondria and prevent the mitochondrial dysfunction [18].

In summary, this study identifies AA is a novel Notch inhibitor, which might be promising for the treatment of sepsis. Furthermore, AA could prevent Notch3 from binding to the IL-6 promoter and regulate mitochondrial function. Our findings provide new insights into the molecular mechanisms of AA-mediated proinflammatory mediator suppression. The novel role of AA may aid in developing better therapeutic strategies for the treatment of proinflammatory disorders.

## MATERIALS AND METHODS

### Reagents and antibodies

LPS from *Escherichia coli* (0111:B4), AA and DAPT were purchased from Sigma–Aldrich. Dll4 was obtained from R&D Systems. β-Actin and Hes1 primary antibody were obtained from Abcam, Notch3 and iNOS primary antibody were from Santa Cruz Biotechnology. Phospho-C-Jun N-terminal kinase (JNK) (Thr183/Tyr185) antibody, phospho-extracellular signal-regulated kinase (ERK)1/2 (Thr202/Tyr204) antibody, phospho-p38 (Thr180/Tyr182) antibody, and phospho-IκBα antibody were purchased from Cell Signaling Technology. All secondary antibodies were from Boster Biological Technology. All other reagents were from commercial suppliers and of standard biochemical quality.

### LPS-induced sepsis in mice and drug administration

Male BALB/c mice, 6–8 weeks of age, were purchased from the Experimental Animal Center, Medical College of Yangzhou University. Animal welfare and experimental procedures were carried out in accordance with the Guide for the Care and Use of Laboratory Animals (Ministry of Science and Technology of China, 2006) and the related ethical regulations of our university. All the animal experiments were made to minimize suffering and to reduce the number of animals used. BALB/c mice were randomly divided into 4 groups of 8: sham control, LPS, LPS+AA (10 mg/kg) and LPS+AA (30 mg/kg). In the LPS+AA groups, AA (10 and 30 mg/kg) was suspended in 0.5% sodium carboxymethyl cellulose (CMC-Na) and administered once daily for 3 consecutive days, while the mice in the sham control and LPS group received an equal volume of CMC-Na. Two hours after the last administration, all mice except the sham control group were administered LPS at 10 mg/kg intraperitoneally. The mice from the sham control group were given an equal volume of PBS and survival was monitored continuously for 48 h.

### Histological analysis

After treatment with LPS for 4 h, livers, kidney and lungs from mice in each group were removed and fixed in 10% formalin, embedded in paraffin, and 5-μm sections were cut from each block and stained with H&E. The stained sections were observed and photographed under a light microscope (200×).

### Assay for serum ALT and blood urea nitrogen BUN

To assay for the serum ALT and BUN levels, blood from mice in each group was extracted from eyeballs and then separated serum by centrifuge at 3500 r/min for 15 min. Serum (50 μl) was mixed with 0.5 ml of ALT or BUN assay solution (NanJing JianChen) and then measured in a spectrophotometer following the supplier’s protocol.

### Cell culture

Murine RAW264.7 macrophages were purchased from Cell Bank of Type Culture Collection of the Chinese Academy of Sciences. Cells were cultured in Dulbecco’s modified Eagle’s medium (Invitrogen) containing 10% fetal bovine serum (Gibco), 100 U/mL penicillin, and 100 mg/mL streptomycin in a humidified atmosphere of 95% air and 5% CO_2_ confluent at 37°C. Before the experiments, the RAW264.7 cells were plated at ∼10^5^ viable cells per well in 6-well plates. Twenty-four hours later, the cells were treated with AA (20–40 μmol/L) and LPS (0.1 μg/mL) for an additional 48 h.

### Assessment of cell viability

After 48 h co-treatment of AA and LPS, The number of cells was determined using a cell counting kit-8 (biyuntian, Nantong, China). 10 μl of CCK-8 was added into medium directly and then the plates were incubated for an 2 h, and the absorbance at 450 nm was measured using a microplate reader (Molecular Devices, Sunnyvale, CA, USA).

### Cellular NO production and cytokines immunoassay

The content of nitrite in the supernatant of cells was detected using the Total Nitric Oxide Assay Kit (Biyuntian). The production of TNF-α, IL-1β and IL-6 from both serum and culture medium supernatant were analyzed using ELISA kits (MultiSciences Biotech) according to the manufacturer’s instructions.

### RNA isolation and real-time polymerase chain reaction (PCR)

For determination of mRNA expression of proinflammatory mediators, Notch receptors and Dll4, total RNA was extracted from RAW264.7 cells using TRIzol reagent (Invitrogen). cDNA was prepared by reverse transcription with oligo (dT) from total RNA extraction. Real-time PCR for Notch signaling molecules and a reference gene (β-actin) were performed in a LightCycler instrument (Roche Molecular Diagnostics) with the SYBR Green Master Mix kit (Takara). Target gene expression was normalized relative to β-actin. PCR primers used in this study are as follows:

**Table:** 

Primers	Gene sequence
β-actin	F: GAAGTCCCTCACCCTCCCAA R: GGCATGGACGCGACCA
IL-1β	F: GAAGTCAAGAGCAAAGTGG R: ACAGTCCAGCCCATACTTT
IL-6	F: CTGATGCTGGTGACAACCAC R: TCCACGATTTCCCAGAGAAC
iNOS	F: GGACCCAGTGCCCTGCTTT R: CACCAAGCTCATGCGGCCT
Notch 1	F: TCAGCGGGATCCACTGTGAG R: ACACAGGCAGGTGAACGAGTTG
Notch 2	F: TGCCAAGCTCAGTGGTGTTGTA R: TGCTAGGCTTTGTGGGATTCAG
Notch 3	F: GGTTCCCAGTGAGCACCCTTAC R: GTGGATTCGGACCAGTCTGAGAG
Notch 4	F: ACCTGCTCAACGGCTTCCA R: AGCTTCTGCACTCATCGATATCCTC
Dll 4	F: GTCCAACTGTGGCAAACAGCA R: AGCATATCGCTGATATCCGACACTC
CSL	F: TCGATGCTAAACGACGTCAC R: TCAATTCCAGAAACCGCTATG

### Western blot analysis

Cells plated in 6-wells were lysed in 100 μL precooling lysis buffer containing 0.5% TritonX-100, 100 mmol/L Tris–HCl, 150 mmol/L NaCl and 0.1 IU/mL aprotinin for 30 min on ice and centrifuged at 12,000 *g*. The total protein was collected from the supernatant and protein concentration measured by protein assay kit (Nanjing Jiancheng Bioengineering Institute). The protein was mixed with double volume of sample buffer (62.5 mM Tris, pH 6.8, 2% SDS, 5% mercaptoethanol, 1% bromophenol blue, and 25% glycerol) and boiled for 5 min. Protein (20 μg) was separated on 12% SDS-PAGE and transferred to a polyvinylidene difluoride membrane. The membrane was blocked with 5% nonfat milk for 1 h at room temperature and incubated with the indicated antibodies. Protein bands were visualized using western blotting.

### Dll4 stimulation

Recombinant mouse Dll4 protein (2.5 μg; R&D) or 2.5 μg IgG (Santa Cruz Biotechnology) as a control were diluted in 1 mL 0.1% gelatin in phosphate-buffered saline. Six-well dishes were coated with 1 mL of this mixture for 12 h at 4°C. The solution was removed and 10^5^ RAW264.6 cells were seeded. RNA was isolated after LPS treatment.

### ChIP

ChIP assay was conducted using the EZ Magna ChIP Kit (Millipore). The presence of DNA fragments extracted from cells was detected by quantitative PCR with IL-6 promoter primers (CSL). Control amplification was performed using DNA input as a reference.

### Flow cytometry for ROS and MMP

ROS levels were measured using the ROS-specific probe 5′,6′-chloromethyl-2′,7′-dichlorodihydrofluorescein diacetate (H2DCFDA, Beyotime Institute of Biotechnology, Nantong, China). Meanwhile, MMP was monitored by 5, 5′, 6, 6′-tetrachloro-1, 1′, 3, 3′-tetraethylbenzimidazolcarbocyanine iodide (JC-1). Briefly, cells after treated with AA and LPS were incubated with 5 μmol/L DCFH-DA in free serum culture medium or 10 μmol/L JC-1 in completed medium for 30 min at 37°C. Finally, the results were analyzed by flow cytometry.

### Luciferase ATP assay

ATP quantification was measured by the Beyotime chemical luciferase ATP assay kit. In brief, cells were harvest after treated and then lysed in a buffer containing 10 mmol/L Tris and 0.05% Triton X-100 before added to the luciferin–luciferase assay mixture for ATP measurement. The luminescence was analyzed by multifuctional microplate reader (Perkinelmer, USA).

### Statistical analysis

Differences were tested by one-way analysis of variance followed by the Student–Newman–Keuls test as a post hoc test. *p* < 0.05 was considered significant. The Kaplan–Meier method was used to evaluate differences in mortality between the groups. All of the experiments were performed in triplicate and repeated 3 or 4 times.

## Abbreviations

LPS: lipopolysaccharide
TNF-α: tumor necrosis factor-α
IL-1β: interleukin-1β
iNOS: intracellular nitric oxide synthase (iNOS)
NF-κB: nuclear factor-κB
MAPK: mitogen-activated protein kinase
RA: rheumatoid arthritis (RA)
Dll) 1: delta-like-1
DAPT: N-[N-(3, 5-difluorophenacetyl)-l-alanyl]-S-phenylglycine t-butyl ester
AA: asiatic acid
CMC-Na: sodium carboxymethyl cellulose
H&E: hematoxylin & eosin
ALT: alanine aminotransferase
BUN: blood urea nitrogen
ChIP: Chromatin immunoprecipitation
MMP: mitochondrial membrane potential
H2DCFDA: 5′,6′-chloromethyl-2′,7′-dichlorodihydrofluorescein diacetate
JC-1: 5, 5′, 6, 6′-tetrachloro-1, 1′, 3, 3′-tetraethylbenzimidazolcarbocyanine iodide
